# A Systematic Phylogenomic Classification of the Multidrug and Toxic Compound Extrusion Transporter Gene Family in Plants

**DOI:** 10.3389/fpls.2022.774885

**Published:** 2022-03-15

**Authors:** Manduparambil Subramanian Nimmy, Vinod Kumar, Backiyarani Suthanthiram, Uma Subbaraya, Ramawatar Nagar, Chellapilla Bharadwaj, Pradeep Kumar Jain, Panneerselvam Krishnamurthy

**Affiliations:** ^1^ICAR–National Institute for Plant Biotechnology, New Delhi, India; ^2^Department of Molecular Biology and Genetic Engineering, Bihar Agricultural University, Bhagalpur, India; ^3^Crop Improvement Division, ICAR–National Research Centre for Banana, Tiruchirappalli, India; ^4^Division of Genetics, ICAR–Indian Agricultural Research Institute, New Delhi, India

**Keywords:** gene family classification, gene family evolution, MATEs, phylogenomics, synteny network, kingdom wide, USEARCH

## Abstract

Multidrug and toxic compound extrusion (MATE) transporters comprise a multigene family that mediates multiple functions in plants through the efflux of diverse substrates including organic molecules, specialized metabolites, hormones, and xenobiotics. MATE classification based on genome-wide studies remains ambiguous, likely due to a lack of large-scale phylogenomic studies and/or reference sequence datasets. To resolve this, we established a phylogeny of the plant MATE gene family using a comprehensive kingdom-wide phylogenomic analysis of 74 diverse plant species. We identified more than 4,000 MATEs, which were classified into 14 subgroups based on a systematic bioinformatics pipeline using USEARCH, blast+ and synteny network tools. Our classification was performed using a four-step process, whereby MATEs sharing ≥ 60% protein sequence identity with a ≤ 1E-05 threshold at different sequence lengths (either full-length, ≥ 60% length, or ≥ 150 amino acids) or retaining in the similar synteny blocks were assigned to the same subgroup. In this way, we assigned subgroups to 95.8% of the identified MATEs, which we substantiated using synteny network clustering analysis. The subgroups were clustered under four major phylogenetic groups and named according to their clockwise appearance within each group. We then generated a reference sequence dataset, the usefulness of which was demonstrated in the classification of MATEs in additional species not included in the original analysis. Approximately 74% of the plant MATEs exhibited synteny relationships with angiosperm-wide or lineage-, order/family-, and species-specific conservation. Most subgroups evolved independently, and their distinct evolutionary trends were likely associated with the development of functional novelties or the maintenance of conserved functions. Together with the systematic classification and synteny network profiling analyses, we identified all the major evolutionary events experienced by the MATE gene family in plants. We believe that our findings and the reference dataset provide a valuable resource to guide future functional studies aiming to explore the key roles of MATEs in different aspects of plant physiology. Our classification framework can also be readily extendable to other (super) families.

## Introduction

A large class of proteins embedded in cell membranes facilitates both the inward and outward passage of cellular solutes based on diffusion or electrochemical gradients. These proteins serve to maintain cell integrity and can be broadly categorized into channels, pumps, transporters, and other carriers (Tang et al., 2020). Multidrug and toxic compound extrusion (MATE) proteins form one of the major multidrug transporter families that intercept the secondary transport of heterogenic cytotoxic compounds using either proton (H^+^) or sodium ion (Na^+^) gradients. These proteins are ubiquitous across all three domains of life and are presumed to have evolved from a common ancestral gene *via* duplication (Omote et al., 2006). The number of MATEs per species across the tree of life is small except for plants, in which the MATE transporter gene family flourished, resulting in an astonishing diversity of functional novelties. Plant MATEs have evolved for the efflux of an array of substrates ranging from xenobiotics and primary (e.g., citrate) as well as specialized metabolites (e.g., alkaloids and phenylpropanoids) to phytohormones (e.g., abscisic acid and salicylic acid), which play significant roles in detoxification (Diener et al., 2001; Li et al., 2002), disease resistance (Nawrath et al., 2002; Ishihara et al., 2008), iron homeostasis (Rogers and Guerinot, 2002), and aluminum tolerance (Magalhaes et al., 2007). MATEs have also been implicated in anther dehiscence and pollen development (Thompson et al., 2010), plant architecture regulation (Li et al., 2014; Suzuki et al., 2015), root development (Upadhyay et al., 2020), organ initiation (Burko et al., 2011), and senescence (Jia et al., 2019).

Classification of genes and proteins based on sequence homology and/or phylogeny into families often provide important indications of the evolutionary past of genes and common ancestry (Frech and Chen, 2010). In a broader sense, MATEs have been classified as one of the four families of multidrug/oligosaccharidyl-lipid/polysaccharide (MOP) superfamilies, members of which may descend from primordial proteins most closely related to prokaryotic polysaccharide transporters (Hvorup et al., 2003). With a limited number of related sequences available, the MATEs of all phyla have been classified into 15 subfamilies (*n* = 203; Hvorup et al., 2003) or three large subfamilies (prokaryotic NorM and DinF, and eukaryotic MATEs) encompassing 14 smaller subgroups (*n* = 861; Omote et al., 2006). Within these classifications, plant MATEs are further clustered into two subfamilies (Hvorup et al., 2003) or one subgroup (Omote et al., 2006).

Advances in sequencing techniques and genome assembly pipelines have led to the availability of hundreds of diverse plant genomes. Consequently, given the significance of MATEs in different aspects of plant growth, development, and defense (Takanashi et al., 2014; Upadhyay et al., 2019), the MATE gene family has been studied extensively in several plant species (Table 1). Based on its phylogenetic topology, the MATE gene family in plants can be classified into several groups and subgroups; however, as such classifications are not systematic, contradictions exist within the literature (Table 1). For instance, Arabidopsis MATEs were assigned to four groups by Wang et al. (2016) compared to five groups by Santos et al. (2017). In other plant species, three to seven phylogenetic groups have been described (Table 1). This confusion is further exaggerated in the case of subgroup assignment. For example, eight (Ia–IIIc) subgroups are assigned to poplar (*Populus trichocarpa*) MATEs (Li et al., 2017) while 10 subgroups (C1-1–C4-3) are assigned to soybean (*Glycine max*) MATEs (Liu et al., 2016). Intriguingly, the naming of groups/subgroups is also inconsistent between published studies (Table 1) and, albeit not without value, existing studies cannot be used to compare the functional and evolutionary relevance of MATEs across plant species. In this context, a systematic classification of the plant MATE gene family is urgently needed.

**TABLE 1:** Discrepancy in the classification of plant MATE gene family.

Plant species	CGS*	Subgroup	References^†^
*Arabidopsis thaliana*	I–IV	/	Wang et al., 2016 ^†^
*Arabidopsis thaliana*	I–V	/	Santos et al., 2017
*Brassica rapa*	1–4	/	Qiao et al., 2020 ^†^
*Brassica juncea*	1–4	/	Qiao et al., 2020 ^†^
*Brassica napus*	1–4	/	Qiao et al., 2020 ^†^
*Brassica nigra*	1–4	/	Qiao et al., 2020 ^†^
*Brassica oleracea*	1–4	/	Qiao et al., 2020 ^†^
*Capsicum annuum*	I–V	/	Chen et al., 2020
*Cicer arietinum*	G1–G4	/	Zhang et al., 2020
*Citrus sinensis*	I–V	/	Juliao et al., 2020
*Glycine max*	C1–C4	C1-1, C1-2, C1-3, C2-1, C2-2, C3-1, C3-2, C4-1, C4-2, C4-3	Liu et al., 2016
*Gossypium arboreum*	MI–MIII	/	Lu et al., 2018
*Gossypium hirsutum*	C1–C4	C1-1, C1-2, C1-3, C1-4, C1-5, C2, C3, C4	Xu et al., 2019
*Gossypium raimondii*	MI–MIII	/	Lu et al., 2018
*Linum usitassimum*	I–IV	/	Ali et al., 2021 ^†^
*Malus* × *domestica*	I–IV	/	Zhang et al., 2021 ^†^
*Medicago sativa*	C I–C IV	C I-1, C I-2, C I-3, C II-1, C II-2, C III-1, C III-2, C III-3, C IV-1, C IV-2, C IV-3	Min et al., 2019
*Medicago truncatula*	I–IV	/	Wang et al., 2017
*Nicotiana tabacum*	I–IV	Ia, Ib, Ic, Id, Ie, IIa, IIb, IIc, III, IVa, IVb	Gani et al., 2021
*Oryza sativa*	1–4	/	Du Z. et al., 2021
*Oryza sativa*	C1–C4	C1-1, C1-2, C2, C3-1, C3-2, C4-1, C4-2, C4-3, C4-4	Huang et al., 2019
*Oryza sativa*	I–IV	/	Wang et al., 2016 ^†^
*Populus trichocarpa*	I–III	Ia, Ib, IIa, IIb, IIc, IIIa, IIIb, IIIc	Li et al., 2017
*Solanum lycopersicum*	I–V	/	Santos et al., 2017
*Solanum tuberosum*	I–IV	/	Huang et al., 2021 ^†^
*Solanum tuberosum*	I–VI	/	Li et al., 2019
*Solanum tuberosum*	I–V	/	Chen et al., 2020
*Vaccinium corymbosum*	I–V	/	Chen et al., 2015
*Zea mays*	I–VII	/	Zhu et al., 2016

*CGS denotes clade, clusters, class, group, and/or subfamily.

*^†^Classification of MATEs in these studies is similar. In each other studies, classification of MATEs is performed by independent phylogenetic observation.*

Many tools have been developed for the automated annotation of protein (super) families (Wu et al., 2003; Frech and Chen, 2010). However, (super) families are routinely further classified into families, subfamilies, and subgroups by manual intervention, and several classifications and nomenclature committees have been established for this purpose. Examples include the committees for aldo-keto reductase (AKR^1^; Hyndman et al., 2003), UDP-glycosyltransferase (UGT^2^; Mackenzie et al., 1997), and cytochrome P450 (CYP450^3^; Nelson, 2009). However, the establishment of such committees for all gene (super) families is not possible, and a systematic user-friendly classification pipeline would be highly beneficial.

Examining the conservation of gene collinearity and synteny provides critical information about the evolutionary history of genomes, gene families, and genes, as well as yielding important insights into the occurrence of ancient polyploidizations, chromosomal rearrangements, gene orthology, and functional novelties (Zhao et al., 2017). Recently, a phylogenomic synteny network and clustering approach was established to uncover and visualize the synteny relationships of target genes across kingdoms of interest (Zhao et al., 2017; Zhao and Schranz, 2017), which outperforms conventional gene family studies in which one or a limited number of species of interest are typically the focus. Synteny network and clustering analyses on a broader scale have also revealed the significance of deep and/or lineage-specific conservation, ancestral translocations, and ancient duplications in the development of functional and phenotypic novelties of certain gene superfamilies, including APETALA2 (Kerstens et al., 2020), auxin response factors (Gao et al., 2020), and type III polyketide synthases (Naake et al., 2021). Such analyses are necessary to unravel the evolution of plant MATE gene families in greater detail.

Here, we report a kingdom-wide analysis of the MATE gene family and synteny network analyses using 74 species from different plant orders. We identify a total of 4,211 MATEs using a hidden Markov model (HMM) profile-based search and classify these into 14 subgroups based on sequence homology and synteny relationships using a four-step process. Following confirmation based on synteny network analyses, we establish a reference sequence dataset for the classification of the MATE gene family in other species. Together with the synteny network clustering analyses, our systematic classification framework reveals several ancient gene duplication and translocation events in the evolutionary history of plant MATE subgroups. We believe that our classification strategy could be effectively applied to other genes (super) families, and our findings provide a valuable resource for ongoing plant MATE research.

## Materials and Methods

### Identification and Characterization of Multidrug and Toxic Compound Extrusion Transporters in 74 Plant Species

We used the genomes of 74 diverse plant species including 39 eudicots, 20 monocots, eight green algae, three basal angiosperms, and a single representative of the following groups: red algae, liverworts, mosses, and lycophytes (Supplementary Table 1). The proteomes of three cucurbit and two banana species were, respectively, downloaded from the Cucurbit Genomics (Zheng et al., 2019)^4^ and Banana Genome Hub (Droc et al., 2013)^5^ databases, and the proteomes of the 69 other species were downloaded from the Phytozome version 13^6^ database (Goodstein et al., 2012). All downloaded proteomes comprised only the primary transcripts. The MatE.hmm file (Pfam No. PF01554.19) corresponding to the HMM of the MATE gene family was downloaded from the Pfam database (version 33; El-Gebali et al., 2019).^7^ The MatE-domain-containing proteins of the 74 plant species were retrieved through “hmmsearch” within the HMMER3 software platform (Mistry et al., 2013), in which the MatE.hmm profile was searched with the gathering threshold (–cut_ga) option against each downloaded proteome. The resulting output files were concatenated, and the amino acid sequences of all hits were interrogated using stand-alone InterProScan (version 5.48-83) software (Jones et al., 2014) to determine their Pfam (Pfam version 33.1) domain composition. The domains were visualized and extracted using the QKdomain pipeline (Bailey et al., 2018).^8^ Intron abundance was calculated using the Genestats pipeline by exploiting the GFF3 files of the corresponding species (Card, 2021).^9^ Protein and domain lengths were inferred using the Seqkt tool (Shen et al., 2016). The presence of signal peptides and transmembrane helices was verified using the SignalP-5 (Armenteros et al., 2019)^10^ and TMHMM version 2 (Krogh et al., 2001)^11^ servers.

### Multiple Sequence Alignment and Phylogenetic Analyses

The full-length amino acid sequences of selected plant MATEs were aligned using MUSCLE version 3.8.1551 with default options (Edgar, 2004). The gappiest positions in the resulting alignment were removed with trimAl version 1.4 using the -gappyout option (Capella-Gutierrez et al., 2009). The trimmed alignment was then used for phylogenetic reconstruction analysis in MEGA X (Kumar et al., 2018), whereby an unrooted neighbor-joining (NJ) tree was generated with 1,000 bootstrap replications by applying the Poisson model, uniform rates, and pairwise deletion options. Concurrently, a maximum-likelihood (ML) tree was generated using the same trimmed alignment in RAxML version 8.2.12 (Stamatakis, 2014) with -m PROTGAMMAAUTO (this option determined JTTDCMUT likelihood as the best protein substitution model for the input alignment and used it for the tree search), -f a (this option would start rapid bootstrap analysis and search for best-scoring ML tree, simultaneously), and 100 bootstrap replications. The resulting phylogenies were visualized and annotated using iTOL v6 (Letunic and Bork, 2021).^12^

### Classification of Plant Multidrug and Toxic Compound Extrusion Transporters

Plant MATEs were classified based on homology and synteny relationships using a four-step process. First, a distance matrix was generated for 3,523 MATEs (which showed comparable protein length and domain architecture to the characterized plant MATEs) based on their protein sequence identity (PSI) using the -calc_distmx command. The resulting distance matrix was utilized for agglomerative clustering using the -cluster_aggd command. Protein clusters sharing ≥ 60% PSI were then retrieved using the minimum linkage (-linkage min) option. These three commands were all executed in the USEARCH version 11 program with default parameters^13^ (Edgar, 2010). Second, a protein database was created with 3,369 MATEs grouped into 14 USEARCH clusters, each with ≥ 50 genes, using ncbi-blast+ version 2.6.0-1 (Camacho et al., 2009), against which all unclassified plant MATEs (*n* = 847) were blasted using the -max_target_seqs 5 and -outfmt 6 options. Subgroups were then assigned to each query based on two conditions: (i) ≥ 60% identity with an e-value threshold ≤ 1E-05 relative to the subject; and (ii) an alignment length covering ≥ 60% of the query’s protein length or ≥ 150 amino acids. Third, if an unclassified query showed a synteny relationship with more than three plant MATEs in the same subgroup, the subgroup of those classified MATEs was assigned to the query. Finally, a new protein database was created based on the MATEs classified during the first three steps against which all remaining, unclassified MATEs were blasted, and subgroups were assigned to the queried MATEs that fulfilled the conditions outlined under step two.

### Genomic Synteny and Network Construction Analyses

To determine the synteny relationships between MATEs across the plant kingdom, genomic synteny, and network construction analyses were conducted for the proteomes of the selected 74 plant species as described in the synteny network (SynNet) pipeline (Zhao et al., 2017).^14^ This was performed using Diamond version 2.0.6 (Buchfink et al., 2015) and MCScanX (Wang et al., 2012). Briefly, Diamond was employed with the “number of hits” option (i.e., -k) set to five for all inter- and intra-pairwise all-vs.-all whole-genome comparisons, in which the proteomes of 74 plant species were blasted with themselves and one another reciprocally (in total 5,476 blasts were made). The resulting blast outputs were then utilized by MCScanX to compute synteny blocks with default parameters (i.e., number of minimum match size for a collinear block = 5 genes; the number of maximum gaps allowed = 25 genes). Followed by, synteny blocks consisting of plant MATEs were extracted from the master synteny file and visualized in Cytoscape version 3.7 (Shannon et al., 2003). Prior to visualization, the MATE synteny file was curated, whereby certain syntenic pairs, having non-MATEs (i.e., a gene in the syntenic pair was not detected during the HMM search), were excluded. Clustering analyses of the synteny networks were performed in the R statistical environment (version 4.0.2), using the “igraph” (Csardi and Nepusz, 2006), “pheatmap” (Kolde, 2015), and “vegan” (Dixon, 2003) packages.

## Results

### Systematic Identification, Classification, and Phylogeny of Plant Multidrug and Toxic Compound Extrusion Transporters

**FIGURE 1:**
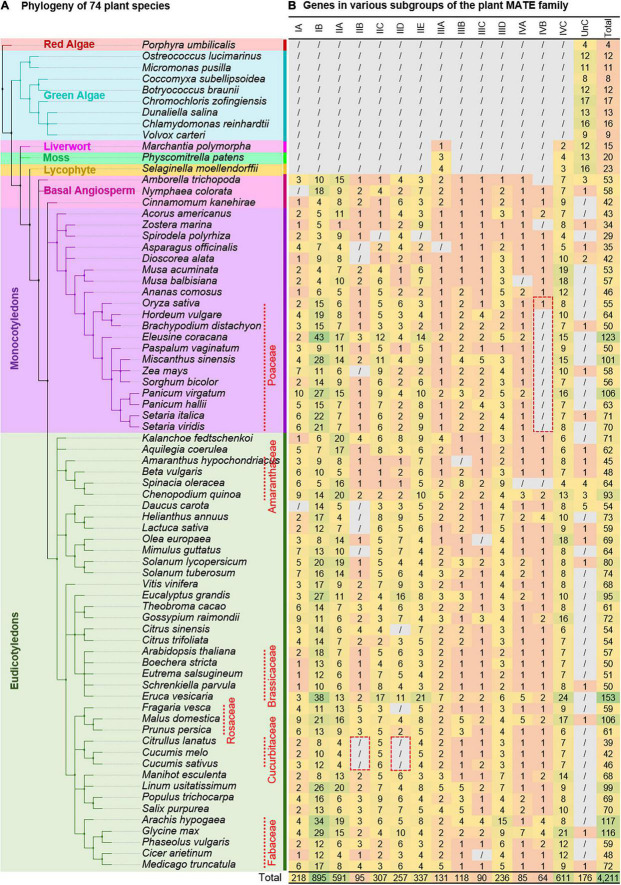
Distribution of multidrug and toxic compound extrusion (MATE) transporters across the plant kingdom. The phylogeny of 74 plant species **(A)** was inferred using the NCBI Taxonomy Browser (https://www.ncbi.nlm.nih.gov/Taxonomy/Browser/wwwtax.cgi). The number of MATEs in different subgroups of each plant genome is shown next to the species names **(B)**. Numbers are colored based on abundance. “/” denotes not detected. Subgroups are assigned based on ≥ 60% protein sequence identity threshold using a set of bioinformatic tools (see section “Systematic Identification, Classification, and Phylogeny of Plant Multidrug and Toxic Compound Extrusion Transporters” for details).

Phylogenomic profiling was performed based on the HMM profile of the MATE gene family (PF01554.19) to explore the complete repertoire of MATEs in the plant kingdom (Supplementary Table 1). An Hmmsearch was conducted separately for each of the 74 plant species to avoid the influence of proteome size/e-value threshold in the MATE detection. A total of 4,217 hits were detected (Supplementary Table 2) with 54 Pfam domains (Supplementary Table 3) and 55 Pfam domain compositions (Supplementary Table 4). While most hmmsearch hits showed two (81.2%) or one (15.9%) MatE domains, few showed non-specific domains in addition to 0–4 MatE domains (1.6%) or multiple MatE domains (i.e., more than two; 0.7%; Supplementary Table 4). We presumed that the existence of non-specific or multiple MatE domains in a single hmmsearch hit resulted from an annotation and/or genome assembly pipeline error. InterProScan did not detect the MatE domain in 0.5% of the hmmsearch hits (Supplementary Table 4), implying that the MatE profile signal was too low in those cases. Gathering thresholds are family-specific bit score thresholds assigned manually by Pfam curators at the time a family is built to exclude any false positive matches (Punta et al., 2012). They are generally considered the reliable curated thresholds defining family membership. Because the hmmsearch was conducted using a gathering threshold option in this study, all of the identified hits were selected to represent the MATE family of plants and were designated as plant MATEs accordingly (Figure 1).

**FIGURE 2:**
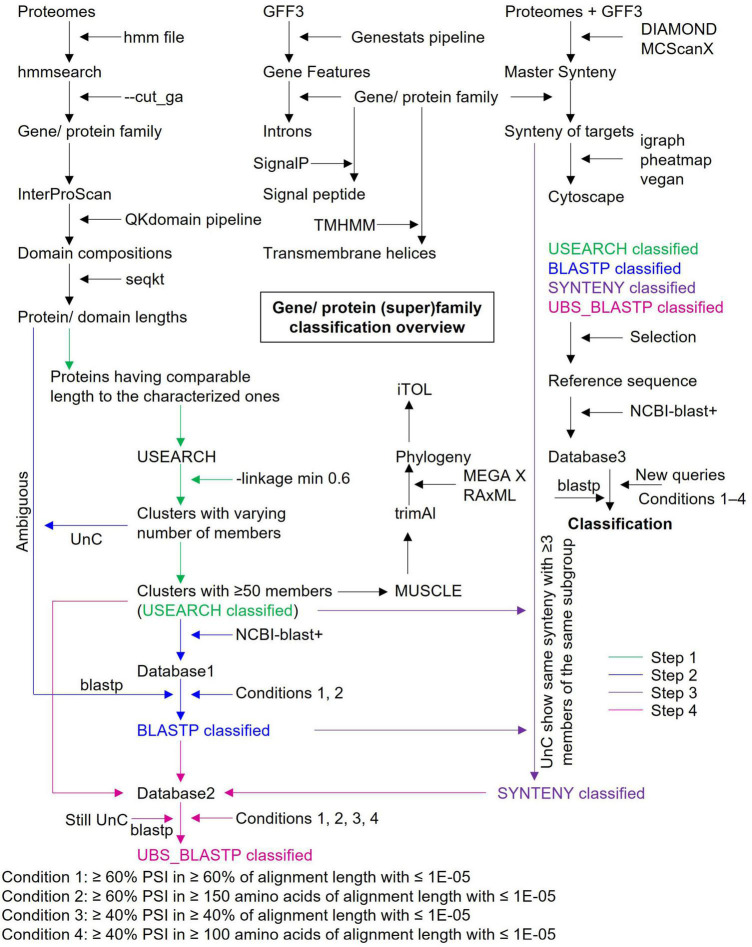
Gene/protein (super) family classification overview. Major steps and bioinformatic tools involved in the classification workflow are highlighted. Reference sequences were selected based on their length and domain architecture from the USEARCH, BLASTP, SYNTENY, and UBS_BLASTP classified genes. UnC, Unclassified. Ambiguous, genes show short-length and/or non-standard domain architectures. UBS, USEARCH, BLASTP, and SYNTENY. The blastp conditions are applied sequentially where appropriate (i.e., condition 1 followed by 2 or condition 1 followed by 2, 3, and 4). The conditions 1/2 and 3/4 are used to determine the subgroups and groups of the gene/protein (super) family, respectively.

Plant MATEs were systematically classified in four subsequent steps using a set of bioinformatics tools by applying a ≥ 60% PSI threshold (Figure 2). The length of all characterized plant MATEs ranged from 370 to 615 amino acids and consisted of one or two MatE domains (Supplementary Table 5). Concurrently, the length of a single MatE domain corresponds to 161 amino acids (PF01554.19). Based on this, and to achieve a better sequence alignment as well as reliable phylogenies, only 3,524 MATEs were selected for the USEARCH cluster analyses, with full-length and MatE domain lengths ranging between 350 and 650, and between 140 and 340 amino acids, respectively (Supplementary Table 6). As the subfamilies of several gene superfamilies are assigned based on the ≥ 60% PSI (for example, the AKR gene superfamily; Hyndman et al., 2003), USEARCH was employed to cluster MATEs with ≥ 60% PSI based on the PSI distance matrix. Two linkage options are available in USEARCH for clustering. First, in the maximum linkage option, members in each cluster share ≥ 60% PSI with every member of the same cluster, while second, in the minimum linkage option, members in each cluster share ≥ 60% PSI with any of one member in the same cluster. Because the maximum linkage option generates many small clusters (data not shown), we applied the minimum linkage option, which identified 121 clusters with 1–718 members (Supplementary Table 7). Fourteen clusters (each comprising ≥ 50 members) were selected to represent the subgroups of the plant MATE gene family that covered 3,369 (out of 3,524) MATEs (i.e., USEARCH-classified MATEs).

**FIGURE 3:**
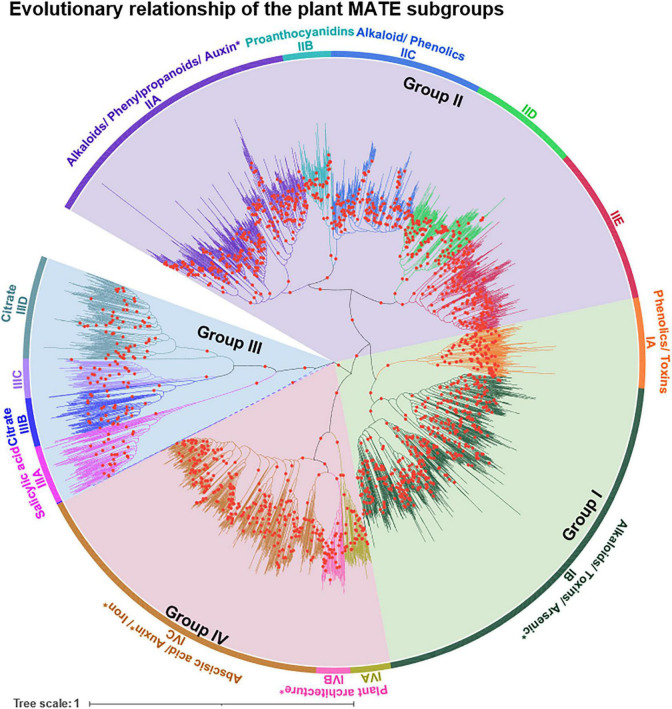
Evolutionary relationship of MATE subgroups in the plant kingdom. By applying ≥ 60% full-length protein sequence identity (PSI) threshold using USEARCH, subgroups were identified for 3,369 MATEs (termed as USEARCH-classified MATEs). USEARCH was employed with a minimum linkage option, meaning that members in each subgroup share ≥ 60% PSI with any single member in the same subgroup. An unrooted neighbor-joining phylogenetic tree was constructed with the trimmed multiple sequence alignment of USEARCH-classified MATEs having 388 amino acid positions. Groups I–IV were designated according to Wang et al. (2016), and subgroups were designated based on their clockwise appearance within each group in the tree. Groups and subgroups are distinguished by different background and branch colors, respectively. All members are clustered according to their subgroup assignment except two, for which branches are indicated by dashes. The functional information of MATE substrates are shown based on Supplementary Table 5. The phylogeny was constructed with 1,000 bootstrap replications in MEGA X and is colored/annotated using iTOL. Bootstrap support values of ≥ 70% were indicated by red circles on tree branches. “*” Exact substrates are not known.

To unravel the evolutionary relationships of the MATE subgroups and to assign logical subgroup names, an unrooted NJ-derived phylogenetic tree was reconstructed using the USEARCH-classified MATEs. Four major phylogenetic groups were identified and named according to Wang et al. (2016), and subgroups were designated based on their clockwise appearance within each phylogenetic group using capital letters (Figure 3). Groups I, II, III, and IV comprised two, five, four, and three subgroups, respectively. The tree topology of the NJ-derived phylogeny was similar to that of the ML-derived phylogeny (Supplementary Figure 1), suggesting that the clustering of subgroups under different methods is reliable.

Next, a BLASTP search was performed to determine the subgroup of unclassified (UnC) MATEs (i.e., the MATEs not used in the phylogeny reconstruction), in which the UnC MATEs were queried against the database of USEARCH-classified MATEs. A subgroup was assigned to 615 MATEs (termed as BLASTP-classified MATEs), which showed ≥ 60% PSI in ≥ 60% of its protein length or ≥ 150 amino acids with an e-value threshold ≤ 1E-05 to the classified MATEs. Subgroup information of USEARCH- and BLASTP-classified MATEs was incorporated into the synteny file. A subgroup of 40 MATEs (termed SYNTENY-classified MATEs) was determined that locate in similar synteny blocks to ≥ 3 MATEs of the same subgroup. A second blastp search was conducted using the classified and UnC MATEs, which identified a subgroup of 11 MATEs based on the stated blastp criteria. The same blastp output was utilized then with a relaxed condition of ≥ 40% PSI in ≥ 40% of its protein length or ≥ 100 amino acids with an e-value threshold ≤ 1E-05 to explore the group of still UnC MATEs. This identified the group of 73 UnC MATEs. Ultimately, six hmmsearch hits were identified as non-MATEs, which did not contain the MatE domain and to which a group/subgroup was not assigned based on any of the criteria described. Excluding these six genes, the number of plant MATEs identified was revised to 4,211 (Figure 1). Overall, the group and subgroup were determined to be 97.5% (4,108 in 4,211) and 95.8% (4,035 in 4,211) of the plant MATEs identified in this study. Intriguingly, a subgroup of 176 hmmsearch hits having MatE domain(s)—the majority of which belong to the algae lineage—was not determined based on our criteria and were tentatively designated as UnC MATEs (Figure 1). The complete attributes of all the identified MATEs are listed in Supplementary Table 8.

To assist the classification of the MATE gene family in other plant species, a reference sequence dataset (termed “RefPlantMATEs”) was prepared with the 3,444 classified MATEs (Supplementary Text 1). Members in the dataset comprised 1–2 MATE domains, and their protein and domain lengths ranged between 351 and 650, and between 79 and 342 amino acids, respectively (Supplementary Table 8). Using this dataset, the MATE gene family of wheat (*Triticum aestivum*; designated as TaMATEs) was systematically classified. Briefly, the 226 TaMATEs identified through hmmsearch, as previously described, were blasted against the RefPlantMATEs protein database, and a subgroup of 224 was successfully determined based on the blastp criteria (Supplementary Table 9). TaMATEs were clustered together with respect to their subgroups in the NJ-derived phylogenetic tree (Supplementary Figure 2), showing the direct benefits of RefPlantMATEs in MATE gene family classification in other plants species. A repository was created in GitHub,^15^ where the step-wise classification of MATEs for additional species is described.

### Genomic Diversification of Plant Multidrug and Toxic Compound Extrusion Transporters

**FIGURE 4:**
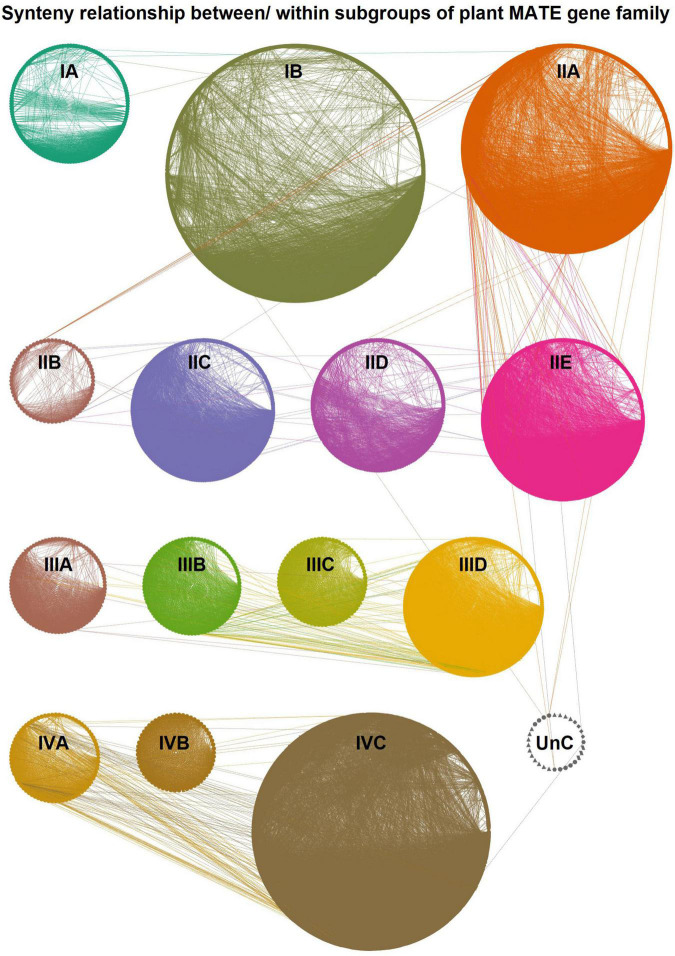
Global synteny network (SynNet) of the plant multidrug and toxic compound extrusion (MATE) transporter gene family. The network was established with all the SynNet relations of plant MATEs within and between genomes that included 3,124 nodes having 55,276 edges and 202 communities. The network was visualized in Cytoscape and the nodes are grouped based on their subgroup. Lines connecting the nodes denote pairwise synteny relationships. Subgroups are highlighted in different colors.

To better understand the evolutionary relationship of plant MATEs, we constructed a synteny network (Figure 4) using the established SynNet pipeline (Zhao et al., 2017; Zhao and Schranz, 2017). Synteny analysis revealed that 74% of the MATEs (3,124 out of 4,211) were located in synteny blocks across and/or within the genomes of the selected 74 plant species (Supplementary Table 10). Most species (*n* = 55) retained ≥ 60% of the MATEs in the synteny blocks (Supplementary Table 11). Synteny conservation was usually greater within the members of the same plant orders/families, likely due to their close evolutionary relationships. For example, 70–90% of the MATEs identified in Amaranthaceae, Brassicaceae, Cucurbitaceae, Fabaceae, and Poaceae species were found in synteny blocks. Intriguingly, certain species, such as barley (*Hordeum vulgare*) and spinach (*Spinacia oleracea*), showed a lower percentage of synteny MATEs compared to other closely related species (Supplementary Table 11), implying that the genome assembly of these species is relatively fragmented.

The synteny MATEs of the 74 plant species (Supplementary Table 10) formed > 50,000 edges (i.e., the number of pairwise connections between MATEs; Supplementary Table 12). Almost all of these connections were intra-group or intra-subgroup types, while only 0.007% and 0.629% were inter-group and inter-subgroup types, respectively (Figure 4 and Supplementary Table 12). On account of connection preponderance, the synteny network was subdivided into 202 synteny communities by igraph, the majority of which retained members of the same subgroups, while 21 communities retained members from different subgroups (Supplementary Table 13). The number of MATEs in each community varied widely between 2 to 213. Three subgroups comprised most communities (i.e., IB–62, IIA–32, and IVC–21), while the other subgroups comprised 3–14 communities (Supplementary Table 14). The percentage of synteny MATEs was relatively low in subgroups IA, IIB, IIC, IID, and IIIA (60%–69%) compared to other subgroups, in which 70–93% of MATEs were retained in synteny blocks.

**FIGURE 5:**
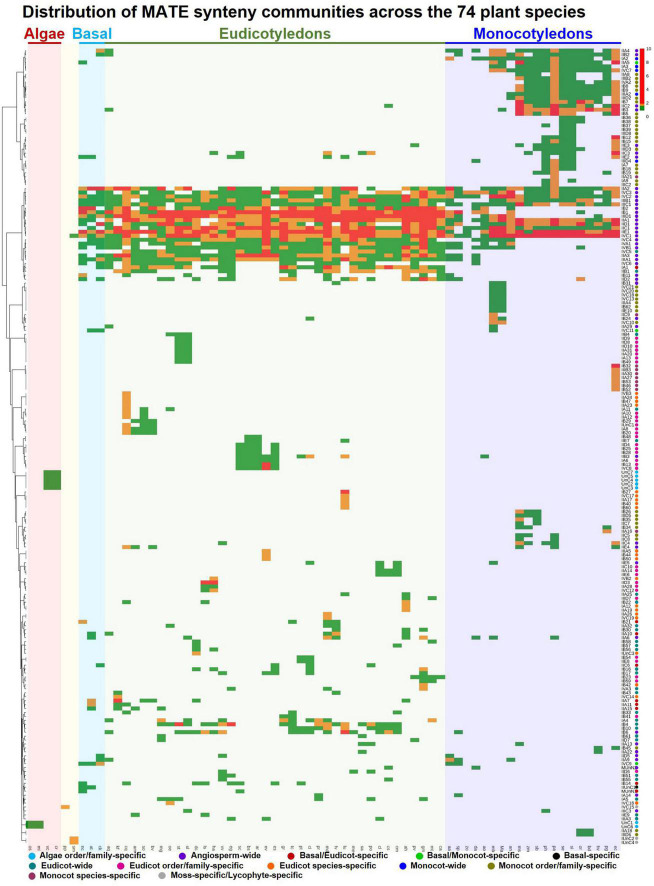
Phylogenetic profile of MATE SynNet communities across 74 plant species. Columns and rows represent plant species and SynNet communities, respectively. The abundance of MATE genes in each community of each species is color-scaled using pheatmap in the R environment. Synteny communities were categorized into 13 types based on their distribution among the 74 plant species. The differently colored circles on the right denote the category assignment (Supplementary Table 15).

A phylogenetic profile was established using the synteny communities of the MATE gene family (Figure 5), in which 13 types of synteny communities were detected based on their species composition (Supplementary Table 15). Four types, namely AFO-specific (members representing two different species of the same order/family of the algae lineage), MoS-specific (members representing a single species of moss), LS-specific (members representing a single species of lycophytes), and basal-specific (members representing two different species of basal angiosperms), corresponded a smaller number of communities and genes (Supplementary Table 16), mainly because of the underrepresentation of those species in our dataset. While three communities of 29 genes were identified as BM-specific (members representing at least one monocot and one basal angiosperm species), nine communities of 95 genes were identified as BE-specific (members representing at least one eudicot and one basal angiosperm species). These BE- and BM-specific communities suggest the occurrence of lineage-specific ancient translocation or genomic rearrangement events during the divergence of basal-eudicot-monocot lineages. Forty-four communities were categorized as angiosperm-wide, with members representing at least one eudicot and one monocot species, and covered 74% of synteny MATEs, showing that most MATEs are located in conserved synteny blocks across angiosperms. More than 51% of the communities (104 out of 202) covering 19% of the synteny MATEs were detected as lineage-specific, with members representing either different orders/families of eudicots (eudicot-wide) and monocots (monocot-wide) or two different species of the same order/family of eudicots (EFO-specific) and monocots (MFO-specific; Supplementary Table 16). Lineage-specific communities suggest that ancient translocation or genomic rearrangement events may have occurred during the divergence of the eudicot-monocot lineages. Species-specific communities (i.e., ES- and MS-specific) corresponded to 15% of the total communities and 2% of the total genes that are usually paralogs generated from local duplication events (e.g., segmental duplication) within a single species.

## Discussion

### Classification Pipeline

Classification of a gene family provides insights into the genetic structure, biological function, and evolutionary trends that are often essential in comparative genomics when tracing the origins and divergence of structural/functional features. Given the key roles of MATEs in different aspects of plant physiology (Takanashi et al., 2014; Upadhyay et al., 2019), the MATE gene family has been studied extensively in several plant species and has been classified into various subgroups (Table 1). As all existing studies have examined MATEs in only a few species and applied classifications based on phylogeny, ambiguity still exists (Table 1), largely due to a lack of reference sequences. To address this issue, we conducted a large-scale phylogenomic study to explore the complete repertoire of plant MATEs, which were then systematically classified using a bioinformatics pipeline involving USEARCH, blast+, and SynNet tools (Figure 2).

Generally, homologous proteins are grouped into families and subfamilies based on their sequence similarity. A threshold of ≥ 40% similarity for families and ≥ 60% similarity for subfamilies are widely implemented for gene superfamilies, such as the AKR (Hyndman et al., 2003), UGT (Paquette et al., 2003), and organic anion transporting polypeptide (Hagenbuch and Meier, 2003) gene superfamilies. Once the plant MATE repertoire was established by hmm profile searches, USEARCH was employed to determine the subgroups of plant MATEs based on a ≥ 60% PSI threshold with the minimum linkage option, and caution was taken during both of these steps. Hmmsearch should be performed for individual species because the size of the proteome influences hit detection. For example, certain hits with low profile thresholds were not detected when the hmmsearch was performed against the master proteome file (in which proteomes of all species were concatenated), but were otherwise detected against the individual proteomes. Likewise, input sequences for clustering in USEARCH are highly valued, and sequences of short-length or non-standard proteins should be avoided (Step 1). For instance, if the input dataset comprises fragment proteins (assuming 50–100 amino acids, which would be very low compared to most members of a target gene family) having considerable similarity to different subgroup members, two or more subgroups would be grouped into one cluster. Another important criterion was the selection of clusters to represent the subgroup/subfamily of a target gene family. Only clusters with a considerable number of members and diverse species representations should be selected. For example, a small group of 13 Brassicaceae MATEs sharing ≥ 60% PSI with one another but not with any of the other members led to their designation as a subgroup (cluster no. 1 in Supplementary Table 7). These, however, exhibited synteny relationships with numerous members of the same existing subgroup (Supplementary Table 12), implying that these 13 MATEs belong to an existing subgroup. Based on this, we selected clusters with ≥ 50 members as MATE subgroups.

To assign logical names to the selected subgroups based on their evolutionary relationships, a family phylogeny was established using NJ and ML methods. The MATE subgroups were clustered under four major phylogenetic clades, which were designated as groups and named according to Wang et al. (2016) (Figure 3). The NJ tree was favored over the ML tree for naming purposes because the former was generated more quickly (1,000 bootstrap replications of 3,369 sequences took ∼10 h with 12 threads) compared to the latter (100 bootstrap replications of 3,369 sequences took ∼10 d with 24 threads). The resulting NJ and ML trees were compared for consistency based on phylogeny topology, and were confirmed to be the same (Supplementary Figure 1). After the USEARCH cluster analysis, a large fraction of the hmmsearch hits were not classified and showed short and/or non-standard domain architectures (collectively termed “ambiguous proteins”). Ambiguous proteins may represent the incompleteness of the genome assembly and/or annotation pipeline, and may obtain their correct sequence structure in forthcoming genome assemblies of the respective species. However, these still require classification to uncover the emergence, expansion, and contraction of the gene family without bias.

Ambiguous proteins were, therefore, blasted against the USEARCH-classified MATE database with an option to output the top five hits (Step 2). Two criteria were implemented in each blastp search to classify ambiguous proteins. The first (i.e., ≥ 60% PSI in ≥ 60% of protein lengths with a 1E-05 threshold) was mainly defined to classify short-length proteins. Because several hits showed multiple MATE domains or non-standard domains, and their amino acid lengths hindered them to satisfy the first criteria, the second criteria were defined (i.e., ≥ 60% PSI in ≥ 150 amino acids of proteins with a 1E-05 threshold). Blastp searches determined subgroups for a large fraction of ambiguous proteins; however, several hits remained unclassified. As gene synteny is evolutionarily more stable than gene sequences (as is the case for the 13 Brassicaceae MATEs previously described), we applied the MATE synteny relationship to the unclassified hits (Step 3). Finally, we generated a further database against which the remaining unclassified MATEs were blasted to determine their subgroup and/or group (Step 4).

All of these steps need to be performed only once for each gene family and, thereafter, a blastp search is sufficient to classify the same gene family in additional species. The fasta header in the reference sequence dataset of the MATE gene family was prepared with five attributes (gene id, species, subgroup, protein length, and classification criteria) separated by pipe (|), which facilitates gene family classification in other species. The usefulness of the reference sequence dataset has already been demonstrated in classifying the MATE gene family of wheat (Supplementary Table 9), and an automated plant MATE classification pipeline is described in the GitHub repository (see text footnote 15). Thus, we believe our approach can be successfully applied to other poorly classified and/or unclassified gene (super) families in the plant kingdom.

### Synteny Network Substantiates the Classification Pipeline

Using the proposed classification pipeline (Figure 2), subgroups were determined for 95.8% (4,035 out of 4,211) of identified plant MATEs (Supplementary Table 8). More than 81% of the unclassified (UnC) MATEs (143 out of 176) belonged to algae and early land plants, highlighting their ancient origins. A similar phenomenon was observed during the classification of CYP450s, in which algae are not amalgamated with higher-order plants (Nelson and Werck-Reichhart, 2011). Thirty-three angiosperm MATEs with MatE signatures did not fulfill any of the classification criteria and, therefore, were designated as UnC. We assume that these proteins correspond to ambiguous sequences resulting from genome assembly/annotation pipeline errors, although may yield classifiable sequences in forthcoming assemblies.

Our subgroup assignment was verified by a synteny relationship given that genes sharing a common ancestry tend to be retained in a similar synteny block across and/or within genomes. Approximately 74% of the plant MATEs (3,124 out of 4,211) were identified as syntelogs (i.e., homologs retained in synteny), meaning that the remaining genes may have lost synteny owing to polyploidization and diploidization, gene translocation, genomic rearrangement, or redundancy in genome assembly/annotation. Alternatively, this may reflect the inability of the SynNet pipeline to detect certain tandem duplicates. This is highlighted using the chickpea (*Cicer arietinum*) MATE gene family (designated as CarMATEs; Supplementary Table 17), in which certain segmental/tandem duplicates with higher PSI were identified as non-syntelogs (i.e., homologs not retained in synteny; Supplementary Table 18).

Plant synteny MATEs formed 55,276 pairwise connections, most of which (99.37%) existed within each subgroup (Supplementary Table 12). This supports the MATE gene family phylogeny established through our proposed classification pipeline. For example, if the MATE phylogeny is considered alone (Figure 3) and sequence homology, as well as synteny, are disregarded, IIB and IIC may be assigned into one subgroup as both show only a marginal distance in the tree. However, both of these subgroups share < 60% PSI, and only 2% of their connections (out of ∼470) were identified as inter-subgroup types (Supplementary Table 12). This implies that our designation of IIB and IIC as separate subgroups is valid. A similar phenomenon is observed in the case of other subgroups, such as IIIB↔IIIC (Figure 3). Overall, only 348 connections were identified as inter-subgroups (Supplementary Table 12), of which few (1–5%) occurred between different subgroups and the majority occurred between IIA↔IIE (20%), IIIB↔IIID (19%), and IVA↔IVC subgroups (35%; Supplementary Table 19). As inter-group and inter-subgroup connections provide evidence for ancient gene duplications followed by lineage-specific gene losses (Gao et al., 2020), the IIA and IIE subgroups may descend from a recent common ancestor. The same logic can be extended to the IIIB/IIID and IVA/IVC subgroups.

### Evolution of the Multidrug and Toxic Compound Extrusion Transporter Gene Family in Plants

All the analyzed plant species contained MATEs, and the total MATE counts per species were twice as high in angiosperms compared to early-diverging land plants (Figure 1), possibly due to polyploidization. Of note, species MATE counts should be considered alongside the genome assembly version (Supplementary Table 1). This is because the number of MATEs we identified for species differs from existing data in some instances, which we believe reflects likely differences in the use of genome assemblies/annotations, selection criteria, and data download sources. For example, our CarMATEs count (*n* = 48) is relatively low compared to that reported by Zhang et al. (2020), who identified 56 CarMATEs using the reference genome of the CDC Frontier available in the NCBI database (BioProject: PRJNA190909). At the same time, compared with previous studies, we identified comparable numbers of MATEs in the dicot model Arabidopsis (*Arabidopsis thaliana*; *n* = 57) and the monocot model rice (*Oryza sativa*; *n* = 55; Huang et al., 2019; Qiao et al., 2020), which further validates our identification strategy based on the MATE-domain based hmm profile search.

Depending on the evolutionary history of the analyzed species, the MATE count in each subgroup varied considerably, and a definitive expansion pattern was observed among them. The expansion was substantial for subgroups IB, IIA, and IVC (each containing > 500 MATEs), moderate for the IA, IIC, IID, IIE, and IIID subgroups (each containing ∼200–300 MATEs), and weak for IIB, IIIA, IIIB, IIIC, IVA, and IVB subgroups (each containing ∼60–130 MATEs). Consistent with the expansion trend, subgroups IB, IIA, and IVC retained a maximum number of synteny communities, whereas those of other subgroups retained only a few (Supplementary Table 14). This suggests that the subgroups IB, IIA, and IVC flourished during plant evolution, with a higher copy-number variation and synteny diversification, while other subgroups evolved more conservatively. For example, AtEDS5 is an essential component of salicylic acid-dependent signaling for disease resistance (Nawrath et al., 2002), while ADS1 (Sun et al., 2011), ZRIZI (Burko et al., 2011), and AtBIGE1A/ZmBIGE1 (Suzuki et al., 2015) are known to be involved in different aspects of plant architecture regulation. AtEDS5 was identified as a member of subgroup IIIA, whereas ADS1, ZRIZI, AtBIGE1A, and ZmBIGE1 were identified as members of subgroup IVC (Supplementary Table 5). Both of these subgroups are evolutionarily most conserved, containing members detected in early land plants through to angiosperms (Figure 1), but followed a contrasting evolutionary path. This implies that the evolutionary trends of MATE subgroups may be associated with the occurrence of functional novelties and/or conserved functions.

Even though we considered all hits of the hmm profile search of the MATE gene family and classified them as fully as possible, several species-specific subgroup losses were observed (Figure 1). We presume that the loss of subgroups in certain species may not be true but may have occurred due to incomplete genome assemblies/annotations in these species. Examples include the loss of IIIA in *Asparagus officinalis*; because the IIIA subgroup was detected in all analyzed species except algae, its loss in Asparagus is highly unlikely. Nevertheless, the following logical observations can be stated based on our results: (i) subgroup IID may have been lost during Cucurbitaceae evolution because its members were not detected in any of the three analyzed genomes of Cucurbitaceae; (ii) subgroup IVB was not detected in any of the 12 Poaceae species (except rice), suggesting its loss during Poaceae evolution; and (iii) the absence of subgroup IIB in three Cucurbitaceae and two Asteraceae species implicates an order/family-specific loss. Notably, members of the subgroup IIB are involved in proanthocyanidin metabolism (Supplementary Table 5), and proanthocyanidins have been detected in cucumber fruit spines (Liu et al., 2019). Therefore, to further verify whether the loss of IIB is true in Asteraceae/Cucurbitaceae, we examined two additional species from Asteraceae and seven additional species from Cucurbitaceae (Supplementary Table 20). Results suggest that the loss of IIB in Asteraceae and the loss of IIB as well as IID in Cucurbitaceae may be true because all the additional species of Asteraceae/Cucurbitaceae lacked the homologs of those subgroups (Supplementary Table 21). This suggests the following two possibilities, of which we favor the former: (i) IIB members may be present in Asteraceae/Cucurbitaceae species, but were not detected in the current assembly versions of the corresponding analyzed genomes; and (ii) biosynthesis/accumulation of proanthocyanidins in Asteraceae/Cucurbitaceae species may have been mediated by members other than those of subgroup IIB.

To unravel structural feature evolution among the subgroups of the MATE gene family, we further examined the RefPlantMATEs dataset given that the inclusion of all MATEs (some of which are possibly redundant or the products of assembly/annotation error) could potentially result in bias. Based on this, the following observations were made (Supplementary Figure 3): (i) the subgroups of groups I/II, III, and IV mostly comprised of 6–10, > 10, and 0–5 introns, respectively; (ii) only a small proportion of all the subgroups (except IIIA) constitute of a single MatE domain, and (iii) the proportions of MATEs with 6–10 TMHMMs was substantial in subgroups IIE, IIIA, IIIB, IIIC, IIID, IVA, and IVB, compared to 11–15 TMHMMs in the other subgroups. These observations suggest that the subgroups of the MATE gene family evolved distinct structural features during the evolutionary history of plants.

### Insights on the Synteny Conservation of Characterized Plant Multidrug and Toxic Compound Extrusion Transporters

Phylogenetic clustering of synteny communities revealed distinct conservation and diversification patterns of genomic contexts in the MATE gene family of plants (Figure 5). Several communities showed deep conservation across the angiosperms, while several other communities showed lineage-, order/family-, and species-specific diversification. Examples were observed in all subgroups at this level. At least 50 plant MATEs have been functionally characterized to date, representing 10 subgroups and 21 synteny communities (Supplementary Table 5). Notably, no MATEs from the subgroups IID, IIE, IIIC, and IVA have been previously characterized, which we believe expands the functional versatility of plant MATEs.

**FIGURE 6:**
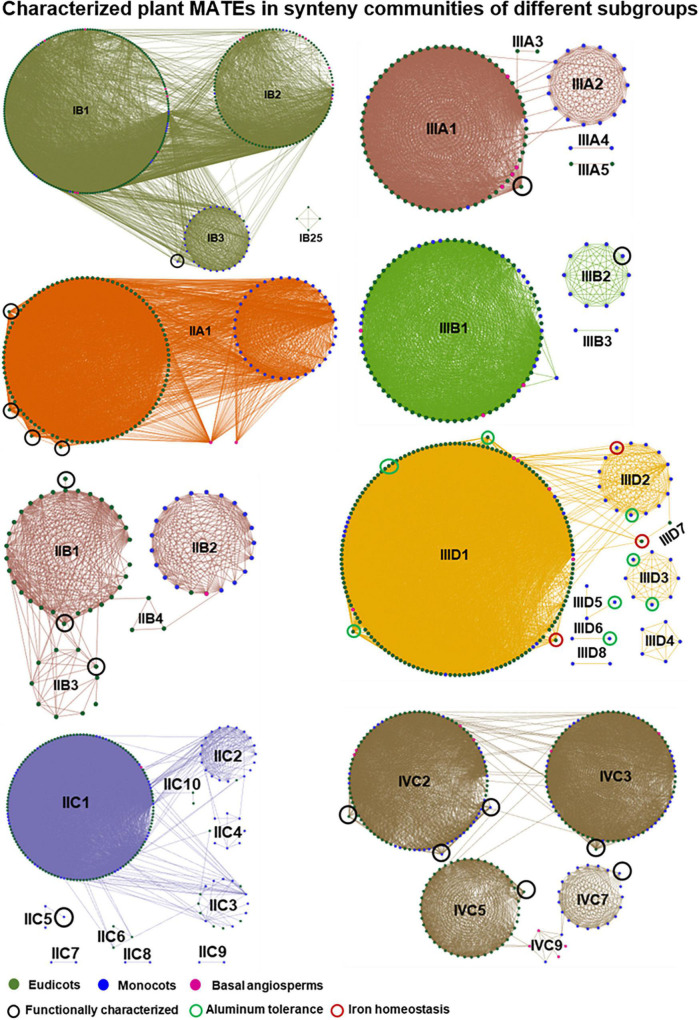
Characterized plant MATE transporters in synteny communities of different identified subgroups. Green, blue, and pink nodes represent different species of eudicot, monocot, and basal angiosperm lineages, respectively. Characterized MATEs were inferred from Supplementary Table 5 and are highlighted with a black circle in all subgroups except IIID. Green and brown circles in subgroup IIID indicate that the MATEs are characterized for aluminum tolerance and iron homeostasis, respectively. Lines connecting the nodes denote pairwise synteny relationships. Subgroups are highlighted in different colors.

Subgroup IA was one of the moderately expanded subgroups with the lowest synteny gene percentage (Supplementary Table 14). AtALF5, conferring toxin resistance (Diener et al., 2001), and AtDTX18, involved in hydroxycinnamic acid amide transport (Dobritzsch et al., 2016), were identified in the Brassicaceae-specific synteny community IA6. IA1 contained members from every eudicot species, except Brassicaceae, and represented the largest IA community (Supplementary Figure 4 and Supplementary Table 15). We presume that a gene translocation event in the Brassicaceae ancestry separated the IA6 community from IA1. Moreover, almost all of the IA communities (except IA14) retained exclusively eudicot or monocot genes (Supplementary Figure 4), indicating an ancient translocation event towards the divergence of the eudicot and monocot lineages. IB was the most diversified subgroup, accounting for the highest gene count and synteny communities (Supplementary Table 14), yet only three of its members were functionally characterized (Supplementary Table 5). Because tobacco (*Nicotiana tabacum*) was not included in our study, we are uncertain whether Nt-JAT1, which is involved in nicotine translocation (Morita et al., 2009), is retained in conserved synteny blocks. OsMATE2, which modulates arsenic accumulation (Das et al., 2018), was located in the IB3 community, which comprised only monocot genes and showed considerable connections to IB1 and IB2 communities (Figure 6). AtDTX1, which mediates the efflux of endogenous and exogenous toxic compounds from the cytoplasm (Li et al., 2002), was present in the Brassicaceae-specific community IB25, which showed no connection to any other IB communities. This suggests a gene transposition event in the ancestral Brassicaceae species.

Subgroup IIA was the third most diversified subgroup, with 73.6% of genes located in 32 synteny communities (Supplementary Table 14), and its members accounted for alkaloid as well as phenylpropanoid transport and auxin homeostasis (Supplementary Table 5). Synteny of Nt-JAT2, involved in nicotine sequestration (Shitan et al., 2014), was not identified, as tobacco was not included in our study. AtDTX30, involved in auxin homeostasis (Upadhyay et al., 2020) was not found in conserved synteny blocks, indicating a species-specific synteny loss. Flavonoid and anthocyanin transporters, namely AtFFT (Thompson et al., 2010), MtMATE2 (Zhao et al., 2011), MTP77 (Mathews et al., 2003), and anthoMATE1/anthoMATE3 (Gomez et al., 2009), were all located in the highly conserved angiosperm-wide community IIA1 (Figure 6). Of note is the presence of large communities (i.e., IIA1–IIA3, each consisting of > 70 genes and > 1,000 connections) with very few inter-community connections and diverse species (eudicot, monocot, and basal angiosperms) compositions (Supplementary Figure 4). This implies that ancient duplication events during the emergence of angiosperms contributed to the expansion/diversification of the subgroup IIA. IIB was one of the most conserved subgroups, with only four synteny communities (Figure 6). MtMATE1 (Zhao and Dixon, 2009), VvMATE2/VvMATE2 (Pérez-Díaz et al., 2014), and AtTT12 (Debeaujon et al., 2001) involved in proanthocyanidin metabolism are all located in the eudicot-wide synteny communities of IIB1 and IIB3. Of note, the IIB2 community comprised only monocot genes, one early eudicot gene, and one basal angiosperm gene that share almost no connections with IIB1/IIB3 (Figure 6). This suggests an ancient gene translocation event during lineage differentiation. Members of the IIC subgroup accounted for nicotine sequestration (NtMATE1 and NtMATE2; Shoji et al., 2009), phenolics efflux (OsPEZ1; Ishimaru et al., 2011), and novel aluminum tolerance mechanisms (ZmMATE2; Maron et al., 2010). While the synteny of NtMATE1 and NtMATE2 is not known and OsPEZ1 lost synteny, ZmMATE2 was identified in a Poaceae-specific IIC5 synteny community. Although IIC showed moderate expansion during plant evolution with 10 synteny communities, inter-community connections were commonly found (Figure 6).

Subgroup IIIA constituted of two major (IIIA1 and IIIA2) and three minor (IIIA3–IIIA5) synteny communities (Figure 6). AtEDS5 is involved in salicylic acid-dependent signaling (Nawrath et al., 2002) and was located in the IIIA1 community, members of which show inter-community connections with IIIA2 and IIIA3, suggesting that genes of IIIA4 and IIIA5 are the products of synteny loss. The MATEs of citrate transporters governing both iron homeostasis and aluminum tolerance (Durrett et al., 2007) represented six of the eight synteny communities in the IIID subgroup (Supplementary Table 5), in which eudicot MATEs show substantial synteny conservation while those of monocots are diversified to a lesser extent (Figure 6). ZmMATE1 (Maron et al., 2010) and OsFRDL2 (Yokosho et al., 2016) were located in two different Poaceae-specific communities, implying loss of synteny events. Since *Lotus japonicus* was not included in our study, we are uncertain whether the nodule-specific LjMATE1 (a member of IIID), which assists the translocation of iron from the root to nodules (Takanashi et al., 2013), is retained in conserved synteny blocks. Recently, ZmMATE6 was identified as contributing to aluminum tolerance through citrate transport (Du H. et al., 2021). ZmMATE6 was a member of the IIIB subgroup located in the Poaceae-specific synteny community (i.e., IIIB2), which had no connection with other communities (Figure 6).

In plant architecture maintenance, AtDETX50, ZRIZI, OsDG1, ZmDG1, ADS1, AtBIGE1A, and ZmBIGE1 have diverse functions, such as organ initiation (Burko et al., 2011), grain filling (Qin et al., 2021), senescence promotion (Jia et al., 2019), and lateral organ-size regulation (Suzuki et al., 2015). These all represent IVC, the second-most diversified subgroup with the second-highest synteny gene percentage (Supplementary Table 14) and five synteny communities (IVC1, IVC2, IVC3, IVC5, and IVC7; Supplementary Table 5). These five communities were found to be partly connected based on one or more genes (Supplementary Figure 4). AtDTX50 involved in abscisic acid transport (Zhang et al., 2014) was located in IVC1, which was conserved very deeply across the angiosperms (Supplementary Figure 4 and Supplementary Table 15). While ZRIZI/OsDG1/ZmDG1 and ADS1 were located in the deeply conserved angiosperm-wide communities IVC2 and IVC3, respectively, the organ initiation and organ size regulators AtBIGE1A and ZmBIGE1 (Suzuki et al., 2015) were located in eudicot-wide IVC5 and monocot-wide IVC7 communities, respectively. Interestingly, IVC5 and IVC7 were interconnected by IVC9 with basal angiosperm genes (Figure 6). This indicates an ancient translocation event during the differentiation of eudicot and monocot lineages. A member of IVB (i.e., At-BIGE1B) has also been reported to mediate the regulation of organ initiation and organ size; however, this gene showed comparably less activity than AtBIGE1A (Suzuki et al., 2015). Inter-community connections involving at least one gene were observed among all the synteny communities of IVC except for IVC13–IVC17, and most of the large communities (i.e., IVC1–IVC4) retained genes from basal angiosperms, eudicots, and monocot lineages. This suggests that expansion/diversification of IVC started before or during the emergence of vascular/flowering plants *via* duplication events.

## Data Availability Statement

The original contributions presented in the study are included in the article/Supplementary Material, further inquiries can be directed to the corresponding author/s.

## Author Contributions

MN, VK, and PK conceived, designed, and performed all the research, and wrote the first draft. BS, US, RN, CB, and PJ contributed to the drafting, revising, and approving of the draft contents. All authors contributed to the article and approved the submitted version.

## Conflict of Interest

The authors declare that the research was conducted in the absence of any commercial or financial relationships that could be construed as a potential conflict of interest.

## Publisher’s Note

All claims expressed in this article are solely those of the authors and do not necessarily represent those of their affiliated organizations, or those of the publisher, the editors and the reviewers. Any product that may be evaluated in this article, or claim that may be made by its manufacturer, is not guaranteed or endorsed by the publisher.

## Footnotes

1
https://hosting.med.upenn.edu/akr/

2
https://prime.vetmed.wsu.edu/resources/udp-glucuronsyltransferase-homepage

3
https://drnelson.uthsc.edu/

4
http://cucurbitgenomics.org/

5
http://banana-genome-hub.southgreen.fr/

6
https://phytozome-next.jgi.doe.gov/

7
http://pfam.xfam.org/

8
https://github.com/matthewmoscou/QKdomain

9
https://github.com/darencard/GenomeAnnotation/blob/master/genestats

10
http://www.cbs.dtu.dk/services/SignalP/

11
https://services.healthtech.dtu.dk/service.php?TMHMM-2.0

12
https://itol.embl.de/

13
https://drive5.com/usearch/

14
https://github.com/zhaotao1987/SynNet-Pipeline/wiki

15
https://github.com/pselva7/PlantMATEsClassification
