# Supplementary material for: A Systematic Phylogenomic Classification of the Multidrug and Toxic Compound Extrusion Transporter Gene Family in Plants
Source: Front Plant Sci. 2022 Mar 15;13:774885. doi: 10.3389/fpls.2022.774885 (PMC8970042; doi:10.3389/fpls.2022.774885)
Supplement: Supplementary file 2 [file Data_Sheet_2.pdf]

(A) Neighbour-joining tree of the plant MATE gene family

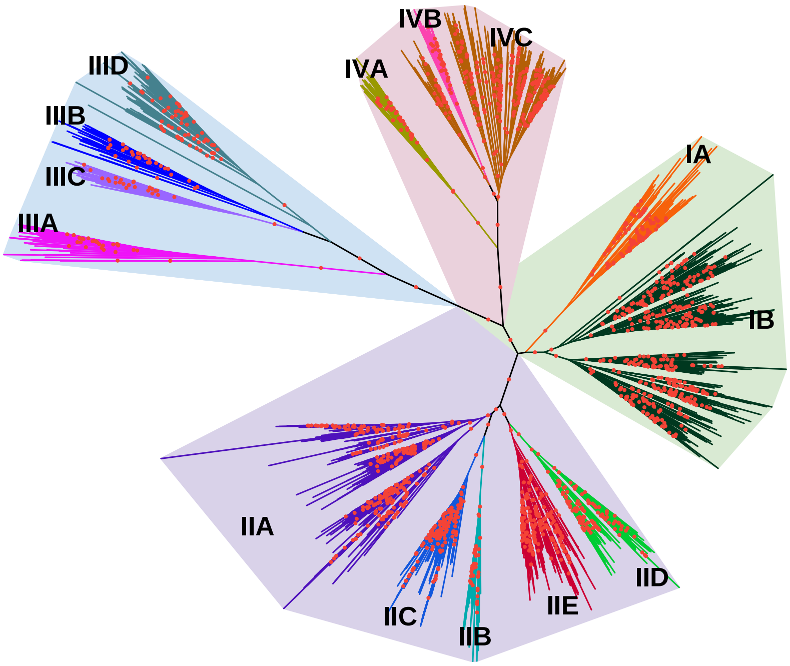

(B) Maximum likelihood tree of the plant MATE gene family

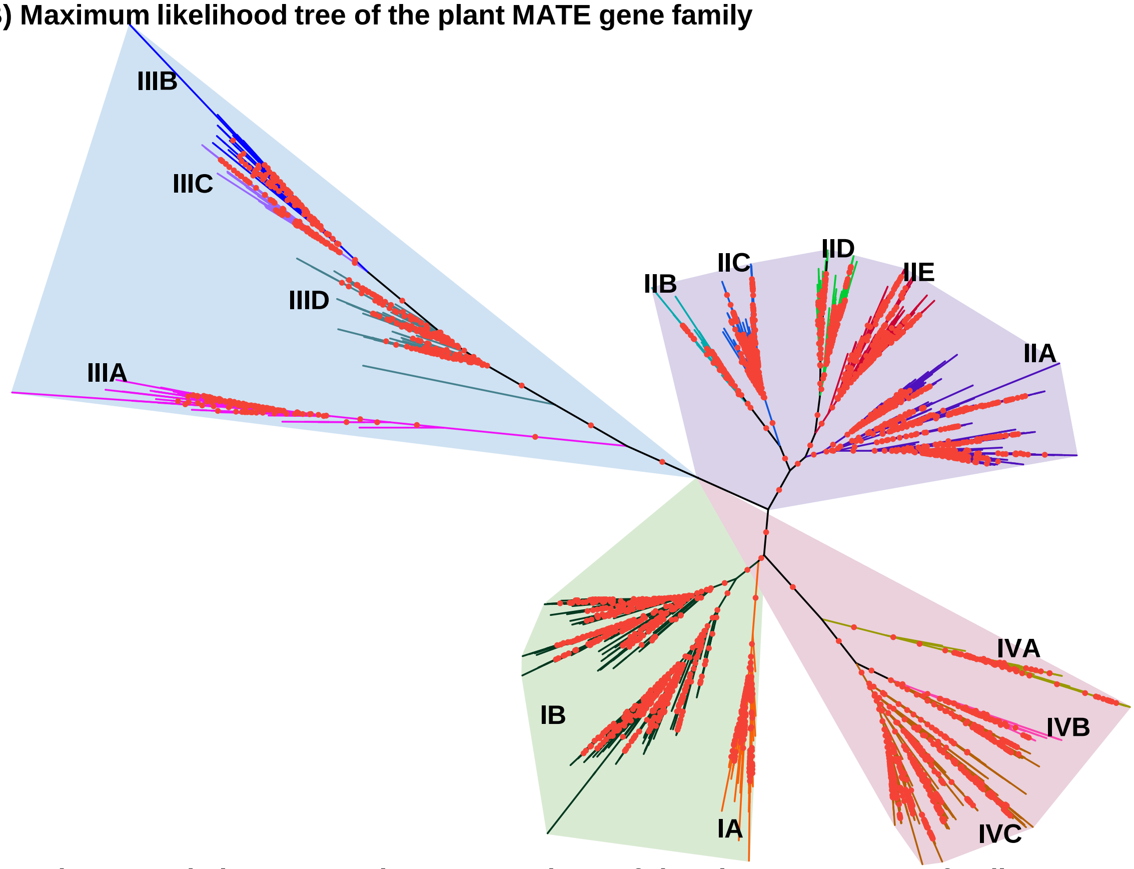

Sup. Figure 1. Phylogeny topology comparison of the plant MATE gene family. Trees were constructed with the trimmed multiple sequence alignment of 3369 USEARCH classified MATEs having 388 amino acid positions. (A) Neighbour-joining phylogenetic tree was constructed in MEGA X with 1000 bootstrap replications using Poisson model, uniform rates and pairwise deletion options. (B) Maximum-likelihood tree was constructed in RAxML (version 8.2.12) with -m PROTGAMMAAUTO, -f a, and 100 bootstrap replication options. Both trees were visualized and annotated using iTOL (https://itol.embl.de/). After phylogeny establishment, two leaves were not clustered into their respective subgroups (similar to Figure 3 in the manuscript) and were removed from both the trees. Assuming group III is evolutionarily the oldest and involving in primary metabolite efflux, both trees were rooted at the clade leading to group III. Bootstrap support values of ≥ 70% were indicated by red circles on branches of both the trees. Groups were highlighted in different backgrounds. Although the clustering order is changed between the trees, all subgroups clustered under the same groups in both trees suggesting that the assignment of MATE groups/ subgroups is reliable.

Phylogeny of the MATE gene family in wheat

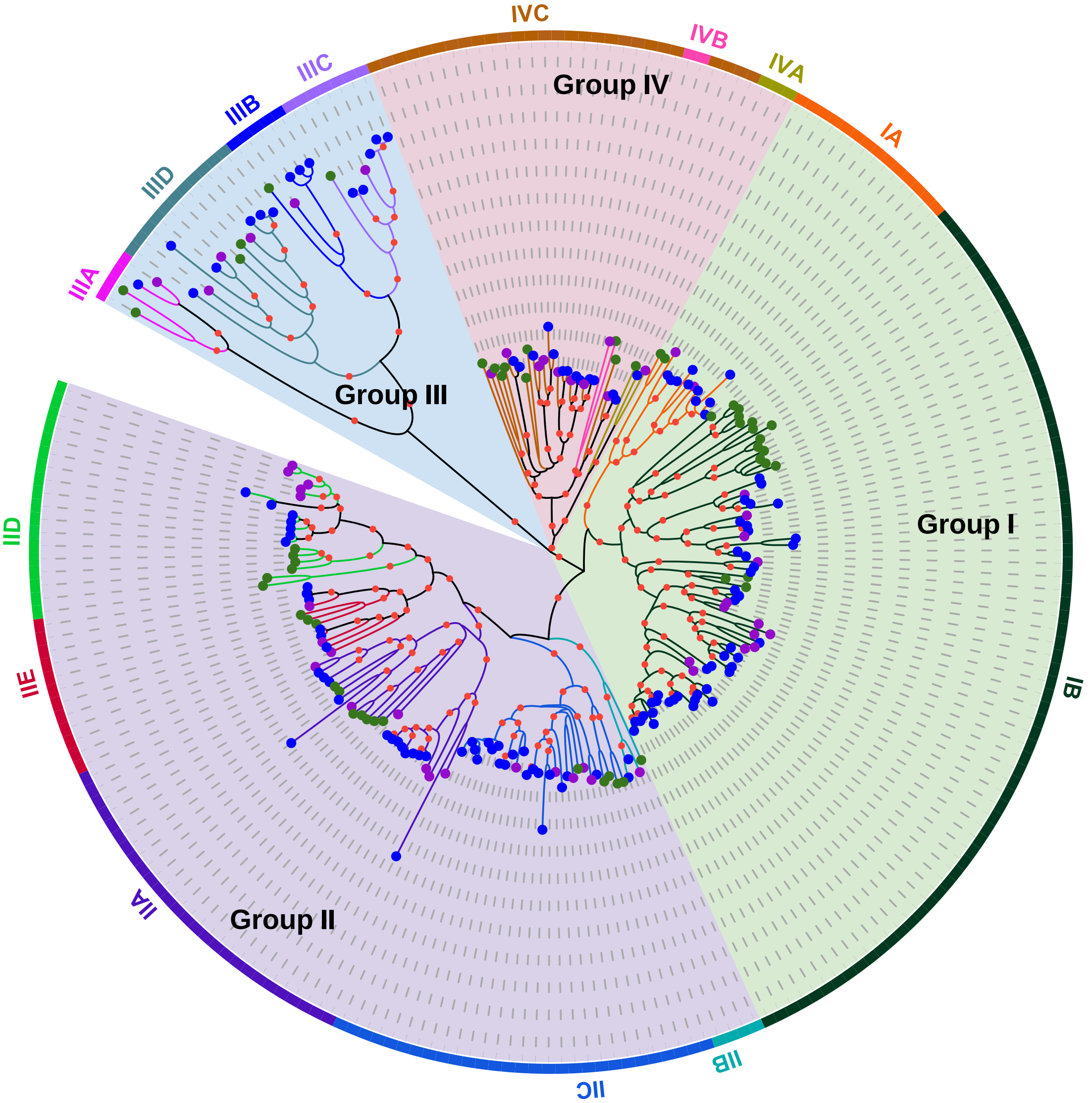

Sup. Figure 2. Phylogeny of the MATE gene family in wheat (*Triticum aestivum*). A neighbour-joining phylogenetic tree is constructed with the trimmed multiple sequence alignment of 236 MATEs having 476 amino acid positions. The aligned dataset includes MATEs from Arabidopsis (n=56), rice (n=51) and wheat (n=129). Tree is generated using MEGA X with 1000 bootstrap replications using Poisson model, uniform rates and pairwise deletion options, followed by visualized/annotated using the online tool iTOL (https://itol.embl.de/). Groups and subgroups, distinguished by different background and branch colours, respectively, similar to Fig. 1 in the manuscript. Leaves highlighted in green, purple and blue coloured circles denote Arabidopsis, rice and wheat MATEs, respectively. Bootstrap support values of ≥ 70% were indicated by red circles on branches of the tree.

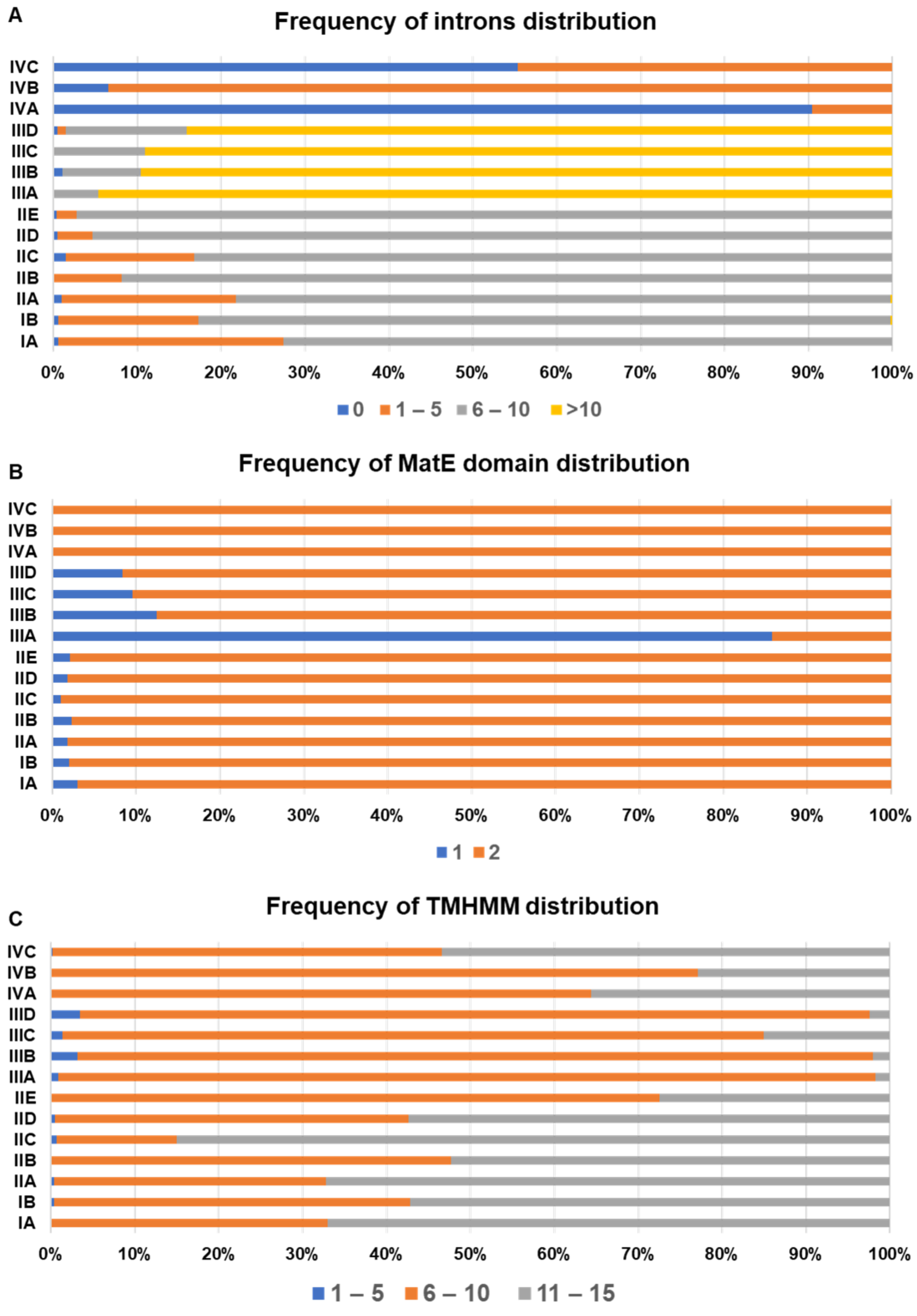

Sup. Figure 3. Structural characteristics of the MATE gene family in plants. Frequency distribution of introns (A), MatE domains (B) and THMHMMs (C) in different subgroups of the MATE gene family in plants. The figure is prepared using the corresponding data of MATEs selected as reference sequences in Table S8.

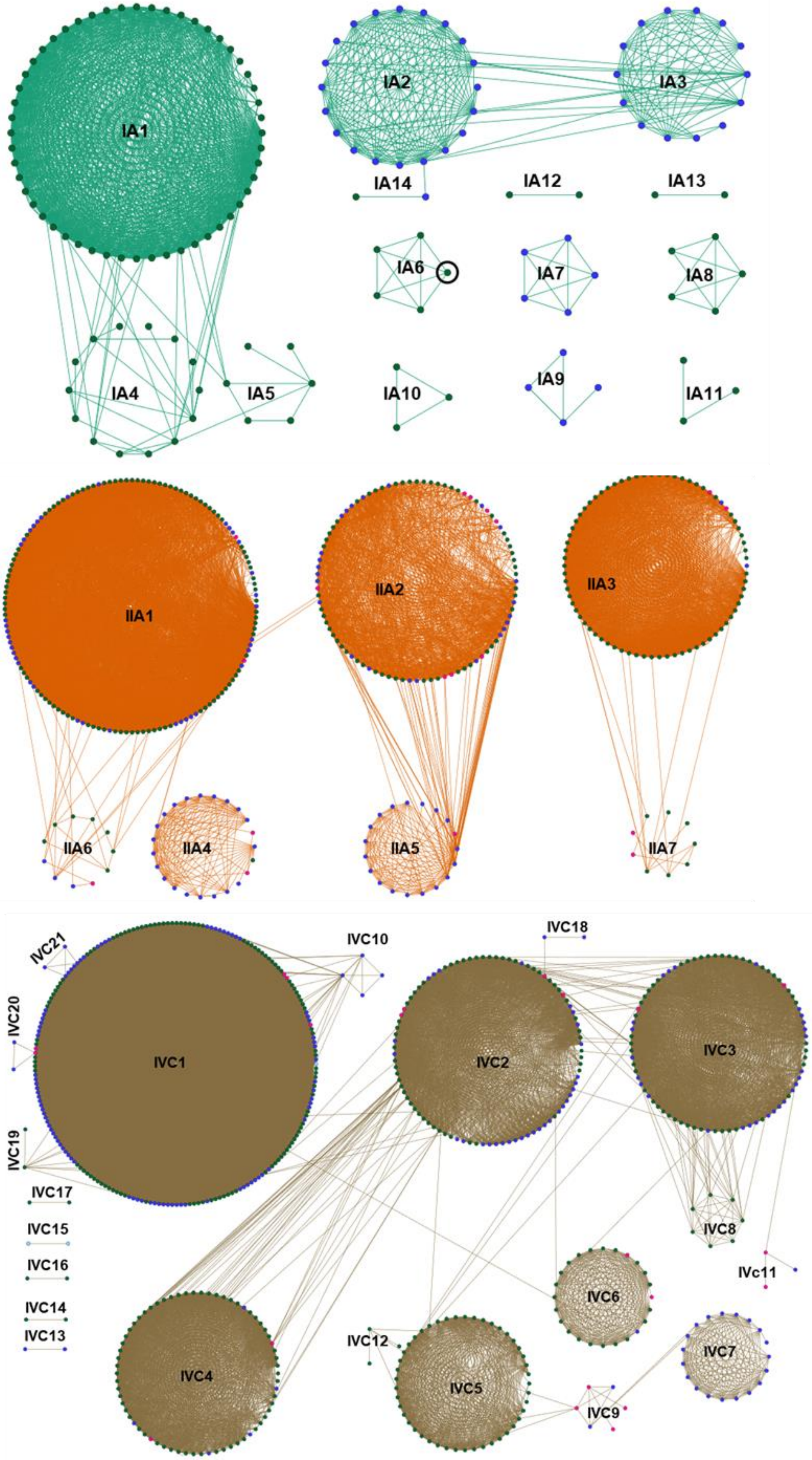

Sup. Figure 4. Highlight of synteny communities from certain subgroups. Nodes filled with green, blue and pink colors represent different species of eudicot, monocot and basal angiosperm lineages, respectively. The figure is prepared in Cytoscape by partioning the global network of plant MATE gene family.
